# Exome Sequencing and MRI in Prenatal Diagnosis: A Case Report of Joubert Syndrome in Colombia

**DOI:** 10.1155/crig/7471200

**Published:** 2026-09-23

**Authors:** Walter Annicchiarico, Leidy Ximena Peña-Pardo, Andrea Muñiz Redondo, Karla Fonseca Cruzate, Mauricio Castro, Gustavo Betin, Jezid Miranda

**Affiliations:** ^1^ Department of Obstetrics and Gynecology, Faculty of Medicine, University of Cartagena, Cartagena, Colombia, unicartagena.edu.co; ^2^ Department of Obstetrics and Gynecology, Clinica de la Mujer, Cartagena de Indias, Colombia; ^3^ Department of Obstetrics and Gynecology, Hospital Serena del Mar and Fundación Santa Fe de Bogotá, Bogotá, Colombia

## Abstract

**Objective:**

To report the prenatal diagnosis of CSPP1‐related Joubert Syndrome 21 (JS21) in a fetus with multiple congenital anomalies, highlighting the complementary role of detailed neuroimaging and molecular genetic testing.

**Methods:**

A fetus with suspected central nervous system anomalies underwent detailed prenatal ultrasound and fetal neurosonography, followed by magnetic resonance imaging (MRI) and exome sequencing. We reviewed prenatal imaging, molecular findings, and postnatal outcomes.

**Results:**

A 25‐year‐old G4P2011 patient with a history of adverse pregnancy outcomes associated with fetal malformations was referred at 32 weeks of gestation. Ultrasound findings included occipital meningocele, cerebellar vermis agenesis, and bilateral cleft lip and palate. Fetal MRI confirmed the characteristic molar tooth sign, raising suspicion of Joubert syndrome. Exome sequencing identified a homozygous canonical splice‐site variant in *CSPP1,* NM_001382391.1:c.2968 + 1G > A (rs587777142), classified as pathogenic according to ACMG/AMP criteria. The pregnancy resulted in the birth of a female neonate weighing 3165 g, and postnatal clinical and imaging evaluation supported the prenatal diagnosis of *CSPP1-*related JS21.

**Conclusions:**

This case illustrates the value of integrating detailed fetal neuroimaging with molecular genetic testing for the prenatal diagnosis of Joubert syndrome. Recognition of characteristic imaging findings can guide targeted interpretation of genetic results, allowing earlier diagnosis, more accurate counseling, and coordinated prenatal and postnatal care.

## 1. Introduction

Joubert syndrome (JS) is a rare congenital neurodevelopmental disorder first described by Marie Joubert in 1969, with an estimated incidence of 1 in 80,000–100,000 live births [1]. The condition is likely underdiagnosed, reflecting its marked clinical and genetic heterogeneity. Although next‐generation sequencing has substantially improved diagnostic yield, a molecular diagnosis remains unidentified in up to 35% of affected individuals [2, 3]. Postnatal manifestations commonly include abnormal respiratory patterns, developmental delay, hypotonia, ataxia, and oculomotor apraxia. Additional manifestations may involve the retina, kidneys, liver, and other organs [1, 4–6].

JS is predominantly inherited in an autosomal recessive manner. Exceptions include OFD1‐related disease, which follows an X‐linked pattern, with affected males harboring hemizygous pathogenic variants, and SUFU‐related JS, which may follow autosomal recessive or autosomal dominant inheritance [6, 7]. In 2014, biallelic loss‐of‐function variants in centrosome and spindle pole‐associated Protein 1 (*CSPP1*) were identified as a cause of ciliopathies within the JS spectrum, establishing *CSPP1* as a JS‐associated gene [8]. *CSPP1* (OMIM #611654) encodes a centrosomal and ciliary protein involved in cell‐cycle progression, spindle organization, and primary cilium formation and function [9]. Biallelic pathogenic variants in CSPP1 cause Joubert Syndrome 21 (JS21; OMIM #615636).

Prenatal diagnosis of JS remains challenging and relies on recognition of characteristic neuroimaging findings followed, when available, by molecular confirmation. Access to advanced genomic testing, particularly prenatal exome sequencing (ES), remains limited and uneven in many middle‐income countries, potentially delaying or preventing a definitive molecular diagnosis. This is particularly relevant for genetically heterogeneous disorders such as JS, in which the prenatal phenotype may not identify the underlying molecular cause. Here, we report the prenatal diagnosis of JS21 in a fetus carrying the homozygous canonical splice‐site variant CSPP1 NM_001382391.1:c.2968 + 1G > A (rs587777142), identified by fetal ES. To our knowledge, this represents the first genetically confirmed prenatal case of this CSPP1 variant reported in Colombia and Latin America. Beyond its molecular characterization, this case highlights the value of integrating expert fetal neuroimaging with genomic testing for the prenatal diagnosis of rare neurodevelopmental disorders in settings where access to advanced genetic testing remains limited.

## 2. Case Presentation

**FIGURE 1:**
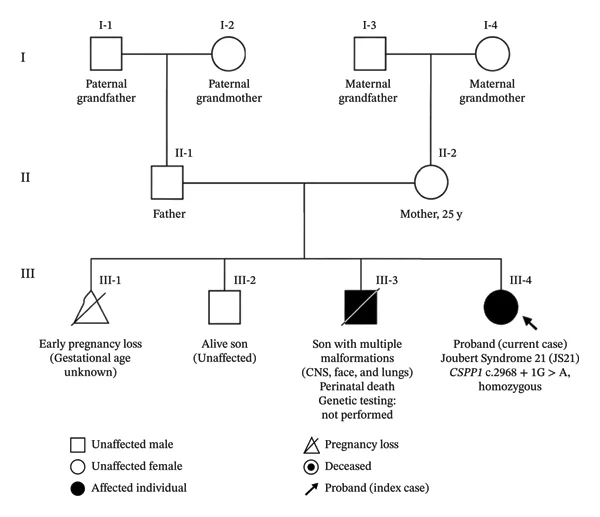
Three‐generation pedigree of the family. The proband (III‐4, arrow) was diagnosed with Joubert syndrome Type 21 (JS21) associated with the homozygous pathogenic variant CSPP1 c.2968 + 1G > A. The couple’s previous pregnancies included an early pregnancy loss (III‐1), a healthy live birth (III‐2), and a previous perinatal death (III‐3) associated with multiple congenital anomalies affecting the central nervous system, face, and lungs, for which genetic testing was unavailable. No parental consanguinity or family history of Joubert syndrome or related ciliopathies was reported.

A 25‐year‐old woman (G4P2011) was referred to the maternal–fetal medicine unit at Clinica de la Mujer, Cartagena, Colombia, for evaluation of suspected fetal central nervous system craniofacial and pulmonary anomalies. Her obstetric history included an early pregnancy loss and a previous perinatal death associated with multiple central nervous system, facial, and pulmonary malformations, for which genetic testing had not been available. The current pregnancy was conceived with the same partner, and there was no known parental consanguinity. Figure 1 shows a three‐generation pedigree.

First‐trimester ultrasound raised suspicion of a central nervous system abnormality, with low screening risk for common aneuploidies. At 18 weeks, detailed anatomical ultrasound demonstrated an occipital meningocele and bilateral cheilopalatoschisis; however, an underlying syndromic diagnosis could not be established. Genetic studies performed earlier in pregnancy, including in situ hybridization (FISH) targeting Chromosomes 13, 18, 21, and the sex chromosomes, were inconclusive because of maternal contamination. The patient was referred to our maternal–fetal medicine unit at 33 + 2 weeks of gestation.

Detailed fetal neurosonography confirmed the occipital meningocele and demonstrated partial agenesis of the cerebellar vermis. Given her recurrent history of fetal malformations and the current complex phenotype, the parents were counseled regarding further diagnostic evaluation. Following informed consent, a repeat amniocentesis was performed for ES, and fetal brain magnetic resonance imaging (MRI) was obtained to further characterize the central nervous system abnormalities.

**FIGURE 2:**
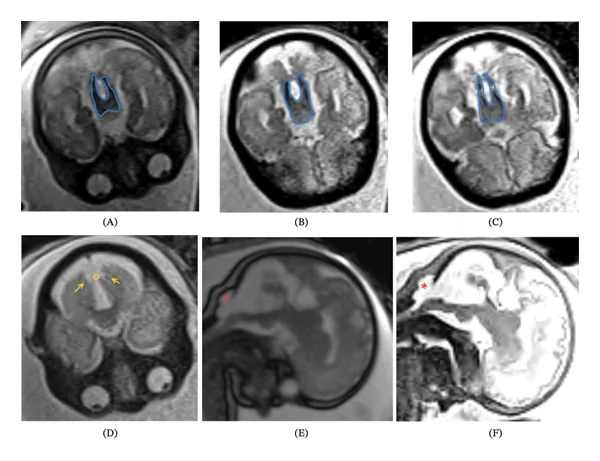
Documented defects in fetal brain MRI: (A, B, C) signs of the “molar tooth” (pathognomonic sign). (D) Agenesis of the vermis of the cerebellum. (E, F) Occipital meningocele.

Fetal MRI was performed using a 1.5 T magnet system with T2‐weighted HASTE sequences acquired in axial, sagittal, and coronal planes. MRI demonstrated a small (approximately 1 cm) occipital meningocele, partial agenesis of the cerebellar vermis, deepening of the interpeduncular fossa, and thickened, elongated superior cerebellar peduncles, forming the characteristic molar tooth sign. Additional findings included a bat‐wing configuration of the fourth ventricle and separation of the cerebellar hemispheres (see Figure 2). Together, these findings raised strong prenatal suspicion of JS.

ES, performed with the Nextera XT kit (Illumina), identified a homozygous canonical splice‐variant in *CSPP1*, NM_001382391.1:c2968 + 1G > A (rs587777142), classified as pathogenic according to ACMG/AMP criteria. The variant substitutes adenine for guanine at the invariant +1 position of the canonical splice donor site and is expected to disrupt normal pre‐mRNA splicing, resulting in loss of *CSPP1* function. This classification was supported by PVS1 (very strong evidence) because the variant affects the invariant donor splice site +1, a canonical splice position predicted to disrupt normal RNA splicing and produce loss of function, which is the established pathogenic mechanism for *CSPP1*‐related JS. PM2 (moderate evidence) was assigned because the variant is absent or extremely rare in population databases, and PP3 (supporting) because multiple computational algorithms consistently predicted a detrimental effect on RNA splicing, as reported in the laboratory analysis. PP4 (supporting) was applied because the fetal phenotype, particularly cerebellar vermian abnormality and the molar tooth sign, is highly specific for JS. Parental segregation analysis was not available; therefore, PP1 was not applied. The imaging and molecular findings supported a prenatal diagnosis of CSPP1‐related JS21. The diagnosis, prognosis, and implications for the current and future pregnancies were discussed with the parents.

The parents elected to continue the pregnancy. Delivery was achieved at term, resulting in the birth of a female infant weighing 3165 g (57th percentile; *z*‐score +0.18), a length of 51 cm (94th percentile; *z*‐score +1.56), and a head circumference of 35 cm (93rd percentile; *z*‐score +1.45), according to the INTERGROWTH‐21st International Newborn Size Standards, with bilateral cheilopalatoschisis. Immediate postnatal care included admission to the neonatal intensive care unit and transfer to a tertiary center equipped for pediatric surgery and neurosurgery. Surgical interventions included occipital meningocele repair, ventriculoperitoneal shunt placement, valve replacement, tracheostomy, and receiving enteral nutrition through gastrostomy. Neurologically, the infant exhibited central hypotonia, a dilated right pupil, decreased reflexes, and persistent flexion of the upper extremities with hands in a supine position. At the time of this report, the patient is 6 months old and remains under supportive and palliative care due to her poor prognosis. Written informed consent for publication of this case was obtained from the parents, and the patient’s data were kept entirely confidential and private. Ethics committee approval does not apply to this case report.

## 3. Discussion

The prenatal diagnosis of JS relies on a combination of advanced imaging techniques, such as detailed ultrasonography, fetal MRI, and ES [10]. Ultrasonography often identifies cerebral and extracerebral anomalies such as increased nuchal translucency, enlarged posterior fossa, agenesis of the cerebellar vermis, midbrain defects, agenesis of the corpus callosum, occipital encephalocele, and ventriculomegaly. However, these findings are nonspecific and insufficient for a definitive diagnosis [2]. Identifying the hallmark sign via ultrasound is challenging without clinical suspicion and specialized expertise [10].

MRI provides a complementary visualization of the “molar tooth” sign. This pathognomonic sign reflects cerebellar vermis hypoplasia, thickened cerebellar peduncles, and a deepened interpeduncular fossa, mimicking a molar tooth [5, 11]. These findings are critical for confirming the diagnosis and are closely associated with peduncular malformations. In addition, ES significantly enhances diagnostic precision by identifying genetic variants linked to JS, of which approximately 40 have been described to date [3, 11]. This underscores the importance of integrating fetal ES into the prenatal diagnostic workflow, particularly when phenotypic anomalies are identified by ultrasound and MRI [10–12].

Previous reports have described the challenge of diagnosing JS prenatally. Lin‐Xi Huang et al. described 13 cases of JS in which prenatal ultrasound, fetal brain MRI, and ES were performed. Their findings highlighted significant variability in imaging features and genetic variants, underlining the complexity of establishing a definitive prenatal diagnosis and the inherent limitations of assessing functional neurological outcomes in fetuses [2, 10].

Despite advancements, the differential diagnosis of JS in the prenatal period remains complex due to overlap with conditions such as Meckel–Gruber syndrome, nonobstructive hydrocephalus, autosomal recessive polycystic kidney disease, and various brain anomalies such as agenesis of the corpus callosum and holoprosencephaly [6, 11]. This highlights the need for advanced prenatal diagnostic techniques, such as MRI and ES, to distinguish JS from other neurodevelopmental disorders. However, in low‐ and middle‐income countries, prenatal diagnosis of conditions such as JS faces additional challenges due to the limited availability of resources; limited access to these technologies often delays diagnosis until postnatal life, when neonatal imaging may reveal the “molar tooth” sign alongside clinical features such as apnea and hypotonia.

The prognosis for individuals with JS is generally poor and is directly related to the compromise of other systems, depending on the subtype of JS (see Table 1). The 5‐year survival rate is approximately 50%, with a percentage of cases resulting in mortality within the first year of life. In addition, the consequences and neurological deterioration in postnatal life are significant, so genetic counseling in couples with affected fetuses can be essential in prenatal and perinatal planning and postnatal care [12]. The association with extracranial malformations will also influence the prognosis [13]. In our case, the infant’s prognosis is compromised by multiple complications related to central nervous system disorders, the need for mechanical ventilation due to respiratory distress, and numerous systemic complications associated with the underlying condition; the multidisciplinary medical team considers the limitation of therapeutic efforts due to the limited benefit of major interventions other than those performed at the time of the patient’s follow‐up.

**TABLE 1:** Description of the characteristics and genetic alterations related to the different subtypes of Joubert syndrome.

Clinical subtypes	Characteristics	Genetic variants	
JS classic	Oculomotor apraxia, respiratory anomalies and polydactyly	Probably associated	*CC2D2A (13%), AHI1, CSPP1, CPLANE1, AHI1, KIAA0586, SUFU*
		Probably associated with fewer reported cases	*ARL13B, ARMC9, B9D1, CBY1, CEP104, CEP120, CEP290, FAM149B1, HY1S1, INPPES, KIAA0556, KIAAA0753, KIF7,MKS1, NPHP1, OFD1, PIBF1, RPGRIPIPL, TCTN1, TCTN2, TCTN3, TMEM138, TMEM216, TMEM231, TMEM237, TOGARAM1, ZNF423*

JS with retinal diseases	Blindness, retinitis pigmentosa	Definitely associated	*AHI1, CEP290*
		Probably associated	*CC2D2A, CPLANE1, CSPP1, INPPSE, MKS1,TMEM67*
		Probably associated with fewer reported cases	*ARL3, ARL13B, ARMC9, CEP41, CEP104, CEP164, IFT172, KIAA0556, KIAA0586, NPHP1, OFD1, PDE6D, POC1B, RPGR1P1L, SUFU, TCTN2, TMEM107, TMEM138, TMEM216, TMEM218, TMEM231, ZNF423*

JS with kidney diseases	Nephronophthisis and cystic kidney with corticomedullary cysts, renal atrophy, and small, scarred interstitial kidneys	Definitely associated	*CEP290, RPGR1P1L*
		Probably associated	*AHI1, CCED2A, INPPSE, NPHP1, OFD1, TMEM138, TMEM216, TMEM231*
		Probably associated with fewer reported cases	*AR13, CEP41, CEP164, CSPP1, KIAAA0753, MKS1, PDE6D, PIBF1, POC1B, IFT172, TCTN3, TMEM216, TMEM218, TMEM237, TOGAEAM1, ZNF423*

JS with liver diseases	Portal hypertension, elevated liver enzymes, recurrent cholangitis, and bleeding from gastroesophageal varices with thrombocytopenia	Definitely associated	*TMEM67*
		Probably associated	*CEP290, CC2D2A, MKS1*
		Probably associated with fewer reported cases	*AHI1, CELSR2, CEP164, CPLANE1, CSPP1, IFT172, INPPSE, KIAA0586, KIAA0753, OFD1, POC1B, RPGRIP1L, TMRM216, TMEM231, TOGARAM1*

JS with oral–facial–digital features	Cleft lip and palate, midline tongue groove, gum (tongue) hamartomas, hypertelorism and micrognathia, polydactyly	Definitely associated	*CPLANE1, TCTN2*
		Probably associated	*IFT172, CSPP1, KIAA0586, CEP120, y KIAA0753* *TMEM216, CELSR2*

JS with neurological features	Encephalocele	Definitely associated	*OFD1, TCTN2, CEP290*
		Probably associated	*CPLANE1, RPGR1P1L, TMEM67*
		Probably associated with fewer reported cases	*ARL138, B9D2, CC2D2A, CSPP1, TMEM138, TMEM216, TMEM218, TMEM237*

JS with skeleton features	Polydactyly	Definitely associated	*CPLANE1, TCTN2*
		Probably associated	*KIF7, MKS1, OFD1, RPGRIP1L, TMEM231*
		Probably associated with fewer reported cases	*AEMC9, B9D2, GBY1, CC2D2A, CCDC28B, CEP41, CEP120, CEP164, CEP290, FAM149B1, UNPPSE, IFT74, KIAA0753, PDE6D, SUFU, TCTN1, TCTN3, TMEM67, TMEM107, TMEM138*

To our knowledge, the homozygous pathogenic variant in the *CSPP1* gene, classified as pathogenic according to the ACMG/AMP criteria [NM_001382391.1 > c2968] + 1G > A, rs587777142], has not been previously described in the literature in the Latin American population. These criteria support classification as pathogenic per ACMG/AMP. A search of publicly accessible genomic databases, including gnomAD (Latin American/mixed subpopulation), ClinVar, and indexed literature, yielded no previous reports of the c.2968G > A variant (p.Gly990Arg) in the Latin American population, nor any published cases of prenatal diagnosis of JS associated with *CSPP1* variants in the region, supporting the claim that this would be the first documented case of prenatal diagnosis in Latin America. Table 2 describes the cases of JS associated with *CSPP1* gene mutations and the molar sign. Although the identification of the homozygous variant in the fetus suggests an autosomal recessive pattern, it was not possible to confirm heterozygous carriers in both parents, which is a limitation of this report. This finding was a key factor in providing genetic counseling to the mother, who had a history of the death of one of her children with multiple malformations, including those affecting the central nervous system, without an associated genetic diagnosis.

**TABLE 2:** Genotype and neuroimaging findings in previously reported *CSPP1*‐related Joubert syndrome cases with a documented molar tooth sign compared with the present prenatal case.

Author and year	*CSPP1* variant(s) (zygosity)	Brain MRI findings (molar tooth sign)	Key clinical features	Reference
Wei et al. (2024)	c.[2325_2326del]; [2936G > T] (compound heterozygous)	MTS, corpus callosum hypoplasia, reduced white matter volume	Microcephaly, generalized tonic–clonic seizures, language regression	Wei C, et al. *Front Pediatr.* 2024; 12:1305754
Akizu et al. (2014)	c.652C > T (p.Gln218Ter) (Homozygous)	MTS, cerebellar vermis dysgenesis, hypoplastic brainstem	Classic Joubert syndrome phenotype; bilateral ptosis	Akizu N, et al. *Am* *J Hum Genet.* 2014; 94(1):80–86
Akizu et al. (2014)	c.2243_2244delAA (homozygous)	MTS, cerebellar vermis dysgenesis	Developmental delay, hypotonia	Akizu N, et al. *Am* *J Hum Genet.* 2014; 94(1):80–86
Bachmann‐Gagescu et al. (2015)	c.1180C > T (homozygous)	MTS, elongated superior cerebellar peduncles	Prenatal diagnosis (20–24 weeks’ gestation); postaxial polydactyly	Bachmann‐Gagescu R, et al. *J Med Genet.* 2015; 52(8):514–522
Shaheen et al. (2014)	c.363_364delTA (homozygous)	MTS present	Strabismus, motor and language developmental delay (Case 9 in the review)	Shaheen R, et al. *Am* *J Hum Genet.* 2014; 94(1):73–79
Tuz et al. (2014)	c.2320C > T (homozygous)	MTS present	Features of Jeune asphyxiating thoracic dystrophy (JATD); optic atrophy	Tuz K, et al. *Am* *J Hum Genet.* 2014; 94(1):62–72
Tuz et al. (2014)	c.[658C > T]; [2527_2528delAT] (compound heterozygous)	MTS present	Liver fibrosis confirmed by biopsy; splenomegaly	Tuz K, et al. *Am* *J Hum Genet.* 2014; 94(1):62–72
Tuz et al. (2014)	c.[652C > T]; [1214G > A] (compound heterozygous)	MTS present	Obesity and abnormal ocular movements	Tuz K, et al. *Am* *J Hum Genet.* 2014; 94(1):62–72
Gana et al. (2022)	c.[950 + 1G > C]; [3205 + 1G > A] (compound heterozygous)	MTS, fourth ventricular dilatation	Prenatal diagnosis (21 weeks of gestation); enlarged kidneys	Gana S, et al. *Am* *J Med Genet C Semin Med Genet.* 2022; 190(1):72–88
Present case	c.2968 + 1G > A (homozygous)	MTS, cerebellar vermis hypoplasia, separation of the cerebellar hemispheres, and occipital meningocele/encephalocele	Prenatal diagnosis (32 weeks of gestation); bilateral cleft lip and palate	Present study

## 4. Conclusions

Prenatal evaluation and management of JS require integrating the findings of central nervous system malformations documented by prenatal ultrasonography, fetal brain MRI, and advanced genetic diagnostic techniques such as fetal ES. This combination of tools offers a comprehensive evaluation that improves diagnostic accuracy and allows optimal prenatal and postnatal care planning for affected patients and their families.

## Funding

There is no funding to declare.

## Conflicts of Interest

The authors declare no conflicts of interest.

## Data Availability

Data sharing is not applicable to this article as no datasets were generated or analyzed during the current study.
